# Optimizing Personalized Care Through Multidisciplinary Management in Rheumatoid Arthritis-Associated Interstitial Lung Disease: A Narrative Review

**DOI:** 10.3390/jpm16050268

**Published:** 2026-05-17

**Authors:** Milica Markovic, Gemma Lepri, Marco Matucci Cerinic, Serena Guiducci

**Affiliations:** 1 Division of Rheumatology, AOU Careggi, Department of Experimental and Clinical Medicine, University of Florence, Via delle Oblate 4, 50141 Florence, Italyserena.guiducci@unifi.it (S.G.); 2 Clinic of Internal Medicine, Clinical Center of Montenegro, 81000 Podgorica, Montenegro; 3 Unit of Immunology, Rheumatology, Allergy and Rare Diseases (UnIRAR), Inflammation, Fibrosis and Ageing Initiative (INFLAGE), IRCCS San Raffaele Hospital, Vita-Salute San Raffaele University, 20100 Milan, Italy

**Keywords:** rheumatoid arthritis, interstitial lung disease, multidisciplinary management, personalized medicine, precision medicine, patient experience

## Abstract

**Background:** Rheumatoid arthritis-associated interstitial lung disease (RA-ILD) is a major extra-articular manifestation of rheumatoid arthritis (RA), contributing substantially to morbidity, mortality, and patient burden. Its diagnosis and management require integration of rheumatologic, pulmonary, and radiologic perspectives. Multidisciplinary approaches may provide a practical framework for implementing personalized medicine by integrating clinical and immunologic heterogeneity into individualized care strategies, although real-world data remain limited. **Objectives:** To synthesize primary studies published between 2015 and 2025 examining diagnostic, organizational, educational, and clinical aspects of RA-ILD within a multidisciplinary care framework. **Methods:** A narrative review of original research was conducted using PubMed and major publisher platforms from 1 January 2015 to 30 November 2025. Search terms included “rheumatoid arthritis,” “interstitial lung disease,” “RA-ILD,” “connective-tissue disease ILD,” “multidisciplinary,” “diagnosis,” “management,” and “patient experience.” Reviews and consensus statements were excluded. **Results:** Fifteen articles underwent full-text review, and five primary studies met all inclusion criteria and were incorporated into the final synthesis. RA-ILD emerged as a condition requiring coordinated interpretation of imaging, pulmonary physiology, and rheumatologic features. Multidisciplinary evaluation may improve diagnostic accuracy, reduce unclassifiable ILD, and support differentiation of autoimmune-related from idiopathic disease. A global assessment of ILD multidisciplinary team practices revealed substantial variability in structures and processes. Patient-reported data demonstrated significant emotional distress and a need for clearer communication and coordinated educational support. **Conclusions:** Effective RA-ILD management depends on collaborative assessment across specialties. Multidisciplinary care may support diagnostic precision and provide a practical framework for personalized medicine by integrating clinical, radiologic, and immunologic heterogeneity into tailored diagnostic and therapeutic strategies.

## 1. Introduction

Rheumatoid arthritis-associated interstitial lung disease (RA-ILD) is increasingly recognized as a major determinant of morbidity and mortality in rheumatoid arthritis (RA) patients. While clinically apparent ILD is diagnosed in approximately 5–10% of patients, HRCT studies reveal that subclinical parenchymal abnormalities may be present in up to 30–50% [1,2,3]. RA-ILD is associated with a significantly increased risk of mortality compared with RA without ILD; furthermore, when a radiological usual interstitial pneumonia (UIP) pattern is present, patient survival further declines to similar values observed in idiopathic pulmonary fibrosis (IPF) [4,5,6].

Beyond its impact on mortality, RA-ILD represents a major source of morbidity, contributing to progressive dyspnea, functional limitation, reduced quality of life, and increased healthcare utilization. The clinical course is highly variable and often unpredictable, with some patients exhibiting indolent disease and others developing rapidly progressive fibrosis despite treatment. This heterogeneity complicates both diagnosis and management, as traditional “one-size-fits-all” approaches fail to capture the complexity of disease behavior. Furthermore, therapeutic decision-making is challenged by the need to balance immunosuppressive strategies targeting inflammatory components with antifibrotic approaches in patients with progressive fibrotic phenotypes [4,5,6].

The radiological spectrum of RA-ILD is heterogeneous. UIP and non-specific interstitial pneumonia (NSIP) represent the most frequently observed patterns, with UIP consistently associated with more rapid functional decline and worse survival [4,7,8]. Established risk factors for RA-ILD include older age, male sex, smoking, high-titer of rheumatoid factor or anti-citrullinated peptide antibodies, and long-standing RA [1,5,9]. Despite these associations, individual disease trajectories vary widely: some patients remain stable for years, whereas others exhibit early, progressive fibrosis despite treatment [6,10].

Accurate diagnosis requires coordinated interpretation of HRCT patterns, serologic findings, pulmonary physiology, and rheumatologic assessment. HRCT represents the gold standard for ILD diagnosis, providing superior sensitivity and pattern recognition compared with radiography [7,8], while pulmonary function testing (PFT)—particularly forced vital capacity (FVC) and diffusing capacity for carbon monoxide (DLCO)—characterizes disease severity and detects early progression [6,11]. Because no single specialty can comprehensively capture the full clinical picture, multidisciplinary evaluation has become a cornerstone of ILD management in general and RA-ILD specifically. Evidence shows that collaborative assessment improves diagnostic accuracy, reduces unclassifiable cases, and aligns management decisions across disciplines [12,13,14]. In this context, a multidisciplinary team (MDT) is defined as a structured collaboration involving pulmonologists, rheumatologists, thoracic radiologists, and, when appropriate, pathologists, with the aim of integrating clinical, imaging, and laboratory data to support diagnostic and therapeutic decision-making.

Despite advances in imaging and pharmacologic therapies, the management of RA-ILD remains complex and fragmented in many clinical settings. Decisions regarding diagnosis, classification, and treatment often require the integration of data from multiple domains, including radiology, pulmonary function testing, serologic profiling, and systemic disease assessment. In the absence of coordinated evaluation, discrepancies between specialties may lead to diagnostic uncertainty, delayed treatment, or suboptimal therapeutic choices. These challenges highlight the need for structured multidisciplinary approaches that can synthesize diverse clinical information and support consistent, evidence-based decision-making.

This narrative review synthesizes original studies published from 1 January 2015 to 30 November 2025 focusing on multidisciplinary elements of RA-ILD care: baseline clinical characteristics, diagnostic influence of rheumatological evaluation, global variability in ILD decision-making structures, and patient perspectives on communication and support. In this context, understanding how multidisciplinary care has been implemented and evaluated in RA-ILD is critical to improving clinical outcomes.

The concept of personalized medicine has become increasingly relevant in the management of complex systemic diseases such as rheumatoid arthritis, where clinical heterogeneity, variable disease trajectories, and diverse treatment responses necessitate individualized approaches. In RA-ILD, this need is even more pronounced, as patients present with distinct radiologic patterns, degrees of inflammatory versus fibrotic involvement, serologic profiles, and comorbidities that directly influence prognosis and therapeutic decision-making. Multidisciplinary care provides a structured framework to operationalize personalized medicine by integrating data across specialties, enabling more precise phenotyping, risk stratification, and treatment selection. In this regard, the multidisciplinary team is not only a diagnostic tool but also a key mechanism for translating disease complexity into individualized patient management strategies. In this context, multidisciplinary care can be viewed as a practical and scalable model for implementing personalized medicine in routine clinical practice.

## 2. Methods

We searched the databases PubMed, MEDLINE, Embase, Scopus, and the Cochrane Library for articles published from 1 January 2015 to 30 November 2025. Search terms included: “rheumatoid arthritis”, “interstitial lung disease”, “RA-ILD”, “connective-tissue disease ILD”, “multidisciplinary”, “diagnosis”, “management”, “HRCT”, “pulmonary function”, and “patient experience”. English-language observational cohorts, prospective multidisciplinary team (MDT) studies, clinical research, and registry-based analyses involving adult patients (aged > 18 years) and addressing multidisciplinary ILD care (with attention to RA-ILD or broader CTD-ILD) were included. Studies were selected based on their relevance to key aspects of multidisciplinary care, including diagnostic contribution, MDT structure, and patient-centered outcomes. Only primary studies providing direct or clearly applicable evidence to RA-ILD or closely related CTD-ILD contexts were included to ensure a focused and clinically meaningful synthesis. Reviews, consensus statements, case reports, pediatric studies, non-peer-reviewed items and non-primary literature were excluded. Given the limited number of RA-ILD-specific studies, selected CTD-ILD and broader ILD-MDT studies were included when their findings were directly applicable to RA-ILD. These data were interpreted cautiously as contextual evidence rather than disease-specific conclusions. Two reviewers (MM and GL) screened titles/abstracts. Full texts were retrieved for potentially eligible studies; data were extracted regarding study design, population, relevance to MDT structure or outcomes, and key findings.

Given the narrative nature of this review, no formal protocol registration, PRISMA-based selection process, or risk-of-bias assessment was performed. This review should therefore be interpreted as a focused narrative review informed by a structured literature search, rather than as a systematic or scoping review. Of the 15 full-text articles reviewed, five primary studies were included because they directly addressed at least one core aspect of multidisciplinary RA-ILD care: rheumatologic contribution to ILD diagnosis, MDT structure, integrated clinical-radiologic assessment, or patient-centered outcomes. The remaining articles were excluded because they were reviews or consensus statements, did not provide primary data, focused on broader ILD or CTD-ILD populations without extractable relevance to RA-ILD multidisciplinary care, or did not address MDT structure, diagnostic contribution, management processes, or patient experience.

## 3. Results

The search identified 210 records; after title and abstract screening, 15 articles underwent full-text review. Five primary studies [15,16,17,18,19] met all inclusion criteria and were incorporated into the final synthesis. These studies were retained because each contributed primary evidence to one of three predefined domains of interest: (1) diagnostic impact of rheumatologic assessment, (2) MDT structure and organization, and (3) patient-centered outcomes and experiences. The limited number of eligible studies underscores the scarcity of primary research specifically focused on multidisciplinary care in RA-ILD. A structured summary of all included studies is provided in Table 1.

Levi et al. [15] conducted a prospective study including 60 patients and showed that rheumatologic assessment reclassified 21% of initially diagnosed idiopathic pulmonary fibrosis as CTD-ILD (including RA (in two patients), systemic sclerosis, ANCA-associated vasculitis, antisynthetase syndrome, and IgG4-related ILD).

Although not limited to RA-ILD, the study by Richeldi et al. [16] conducted the first global characterization of multidisciplinary ILD decision-making structures. Responses from 457 unique centers across 64 countries were included in the analysis. The study reported that compared with non-academic and academic non-ILD centers, ILD academic centers reported a higher ILD caseload, held more formal MDT meetings, and were more likely to include histopathology and rheumatology specialists in their diagnostic team.

The observational study conducted by De Lorenzis et al. [17] analyzed 151 consecutive cases that were discussed in an MDT including rheumatologists for a suspected or definite ILD secondary to SARDs, at a large Italian tertiary care university hospital. They computed the agreement between the pulmonologist and rheumatologist in the identification of red flags for systemic autoimmune rheumatic diseases (SARDs) of 81 ILD cases and between the rheumatologist alone and MDT in the confirmation of 70 suspected SARD-ILD progressions. After rheumatological evaluation, the MDT diagnosed in 54 patients (66.7%) a SARD-ILD (RA-ILD was diagnosed in 5 (6.2%). Among the 70 patients, the progression of ILD related to SARD was considered to be certain or probable for 37 patients (52.9%).

Kim et al. [18] using a multidisciplinary collaborative approach designed and established a prospective cohort study of RA patients at a single center to compare the long-term prognosis between RA-ILD patients and RA patients without ILD (RA-non ILD) and to explore prognostic factors among RA-ILD patients. A total of 148 RA-ILD and 410 RA-non ILD patients were enrolled in the study. The chest CT scans of all patients were evaluated by radiologist and rheumatologist.

Dubey et al. [19] explored patient perspectives in 64 RA-ILD patients across 6 different UK socioeconomic areas. Results from this study suggest that enhanced patient support is needed, and this necessitates more effective integration and utilization of the MDT, including specialist nurses, psychologists, pharmacists, and other allied health professionals.

## 4. Discussion

Although based on only five primary studies, the available evidence reveals several key themes that shape contemporary multidisciplinary management of RA-ILD. These findings underscore that RA-ILD cannot be approached as a uniform disease entity, but rather requires individualized assessment strategies consistent with the principles of personalized medicine.

Levi et al. [15] evaluated the contribution of systematic rheumatologic assessment in patients presenting with ILD and demonstrated that structured rheumatologic evaluation frequently uncovered autoimmune features—serologic, clinical, or both—that were not identified during initial assessment. Consequently, a substantial proportion of ILD cases initially labeled idiopathic were reclassified as autoimmune, including cases ultimately attributable to rheumatoid arthritis. Notably, rheumatologic revision established a new diagnosis of RA in two patients and could retrospectively have prevented several invasive procedures. This study provides foundational evidence that early rheumatologic involvement is essential to avoid diagnostic misclassification and ensure accurate identification of RA-ILD.

Expanding beyond individual diagnostic encounters, Richeldi et al. [16] conducted the first global characterization of MDT practices in ILD across institutions worldwide. Marked heterogeneity was observed in team composition, case discussion processes, radiologic interpretation, and documentation of diagnostic conclusions. Importantly, rheumatology involvement was inconsistent despite growing recognition of autoimmune ILD. Although not specific to RA-ILD, these findings underscore the need for structured and standardized MDT frameworks to ensure diagnostic consistency in diseases that require integrated pulmonary–rheumatologic interpretation.

Further reinforcing the importance of rheumatology within MDT structures, De Lorenzis et al. [17] demonstrated that inclusion of rheumatologists significantly improved diagnostic confidence, refined ILD classification, and reduced the proportion of unclassifiable cases. Rheumatologic input proved particularly influential in cases with subtle autoimmune features or atypical radiologic patterns—clinical scenarios in which RA-ILD is especially challenging to identify. Together with the findings of Levi et al. [15], this study strengthens the argument that rheumatology participation is not optional but central to accurate RA-ILD diagnosis.

Kim et al. [18] provided the only prospective cohort study among the included works, offering detailed clinical, serologic, physiologic, and radiologic characterization of RA-ILD. Compared with RA patients without ILD, those with RA-ILD were older, had longer disease duration, were more frequently seropositive, and exhibited higher levels of systemic inflammation. Baseline pulmonary function showed reduced FVC and DLCO, and HRCT demonstrated a heterogeneous spectrum of inflammatory and fibrotic abnormalities. These findings highlight the systemic and multifaceted nature of RA-ILD and illustrate why integrated interpretation of rheumatologic, pulmonary, and radiologic data is indispensable. The complexity of disease expression supports the rationale for multidisciplinary assessment.

These observations directly align with the principles of personalized medicine, as they illustrate that RA-ILD is not a uniform entity but a spectrum of disease phenotypes requiring individualized interpretation and management. Variability in radiologic patterns, inflammatory burden, and systemic disease activity necessitates tailored therapeutic strategies rather than a one-size-fits-all approach. In this context, multidisciplinary evaluation enables the integration of these heterogeneous data points, supporting patient-specific decision-making regarding immunosuppression, antifibrotic therapy, and monitoring strategies.

The most recent study, Dubey et al. [19], expanded the perspective by examining patient-reported experiences across multiple UK centers. Participants described significant psychological distress following RA-ILD diagnosis, often related to difficulty understanding medical terminology and perceived fragmentation of communication between specialists. Many patients reported inadequate explanations of HRCT findings, pulmonary function results, prognosis, and treatment rationale. A recurring theme was the desire for coordinated communication and structured educational support. These findings suggest that MDT structures play a critical role not only in diagnostic accuracy but also in aligning communication across specialties, thereby addressing patient-reported gaps in understanding, reducing uncertainty, and supporting shared decision-making.

These patient-centered insights align with broader evidence from idiopathic pulmonary fibrosis (IPF) and other fibrosing ILDs, where the MDT model has become the diagnostic standard. International IPF guidelines endorse multidisciplinary evaluation as the reference approach, acknowledging that agreement among individual specialists is limited when clinical, radiologic, and pathologic data are interpreted separately [20,21]. Seminal work by Flaherty et al. demonstrated that integration of pulmonologists, thoracic radiologists, and pathologists within an MDT significantly improves diagnostic confidence and interobserver agreement, particularly in distinguishing UIP from alternative patterns [22]. Multicenter analyses by Walsh and colleagues further confirmed that MDT evaluation enhances diagnostic accuracy and reduces variability in HRCT interpretation compared with single-specialist assessment, especially in cases with probable or indeterminate UIP patterns [13].

In IPF, the MDT model has therefore evolved into a de facto standard of care, influencing not only diagnostic classification but also therapeutic decision-making, including antifibrotic initiation and referral strategies [21,23]. The radiologist–pulmonologist interface is central to this process, as structured HRCT pattern recognition must be interpreted within clinical context and pulmonary function trajectories. This collaborative framework is directly applicable to RA-ILD, where differentiation between UIP and NSIP patterns, exclusion of drug-related toxicity or infection, and identification of subtle autoimmune features require integrated expertise. The IPF experience thus provides empirical validation and a practical framework supporting structured MDT pathways in RA-ILD.

Importantly, the MDT model in ILD also serves as a platform for implementing precision medicine approaches. By combining radiologic pattern recognition, serologic profiling, and longitudinal functional assessment, MDTs facilitate more refined disease phenotyping and prognostic stratification. This, in turn, allows clinicians to tailor treatment strategies according to individual risk profiles, such as selecting immunomodulatory therapies in inflammatory-dominant disease or prioritizing antifibrotic approaches in progressive fibrotic phenotypes. Thus, MDT-driven care may serve as a pragmatic bridge between traditional clinical practice and emerging precision medicine paradigms, although this concept requires prospective validation.

Taken together, the included studies illustrate a progressive evolution in the understanding of multidisciplinary RA-ILD care. Initial evidence demonstrated that rheumatologic involvement prevents diagnostic misclassification, followed by recognition of global variability in MDT structures and empirical confirmation that inclusion of rheumatology improves diagnostic confidence. Prospective cohort data subsequently clarified the systemic nature of RA-ILD and the necessity of integrated interpretation, while patient-reported outcomes emphasized the importance of coordinated communication and support.

A critical appraisal of the included studies indicates that the overall strength of evidence remains limited. All five studies were observational or descriptive in design, and none directly compared MDT-based care with non-MDT care or evaluated hard clinical outcomes such as survival, long-term pulmonary function decline, hospitalization, or treatment response. The diagnostic studies support the value of rheumatologic input but rely mainly on reclassification and diagnostic confidence as surrogate outcomes. The global MDT survey provides important organizational insight but is subject to self-reporting bias and does not assess patient-level outcomes. The prospective RA-ILD cohort offers valuable clinical characterization but was not designed to test the effectiveness of MDT care. Finally, the patient-experience survey highlights unmet educational and communication needs, but its findings are based on self-reported perceptions. Therefore, the available literature should be interpreted as supportive but indirect evidence for MDT-based RA-ILD management.

Collectively, these findings suggest that RA-ILD is a multisystem disease that likely benefits from collaborative expertise, although current evidence remains limited and largely observational.

The small number of studies included reflects the significant gap in the current evidence base. While multidisciplinary care is widely recommended in ILD, few studies have specifically evaluated its implementation, structure, or outcomes in RA-ILD populations. This limitation highlights the need for dedicated research in this area and suggests that current clinical practice is largely guided by extrapolation from broader ILD or CTD-ILD data rather than disease-specific evidence.

From a broader perspective, multidisciplinary management may represent a practical platform for advancing personalized medicine in RA-ILD. By integrating expertise across specialties and aligning clinical, radiologic, and patient-reported data, MDTs may facilitate a more nuanced understanding of disease behavior at the individual level. Future research should focus on incorporating biomarkers, genetic data, and digital health tools into MDT frameworks to further refine patient stratification and optimize individualized care pathways.

## 5. Conclusions

The available primary literature suggests that RA-ILD is a multisystem disease whose diagnosis and management may benefit from coordinated expertise across rheumatology, pulmonology, radiology, and pathology. Multidisciplinary evaluation appears to improve diagnostic confidence, reduce misclassification, and enhance interpretation of HRCT findings and pulmonary physiology. Patient-centered research also highlights the need for coherent communication and structured support. However, current evidence remains limited, observational, and largely based on surrogate outcomes rather than direct clinical endpoints. Multidisciplinary care may provide a practical framework for personalized medicine in RA-ILD by integrating clinical, radiologic, and immunologic heterogeneity into individualized diagnostic and therapeutic strategies. Future studies should prospectively evaluate MDT-based models and incorporate biomarkers, digital tools, and advanced phenotyping approaches to further refine precision care.

## Figures and Tables

**Table 1:** Summary of Included Studies on Multidisciplinary Management in RA-ILD.

Study	Design & Setting	Population	Main Findings	Relevance to Multidisciplinary RA-ILD Care
Levi et al., 2018 [15]	Diagnostic impact study with rheumatologic assessment	Patients evaluated for ILD	Rheumatologic exam reclassified many cases as autoimmune ILD.	Prevents misclassification of RA-ILD as idiopathic ILD.
Richeldi et al., 2019 [16]	Global analysis of ILD multidisciplinary structures	Institutions worldwide	Marked heterogeneity in team composition and decision pathways.	Shows the need for standardized multidisciplinary processes.
De Lorenzis et al., 2020 [17]	Multidisciplinary ILD diagnostic evaluation	CTD-ILD and suspected autoimmune ILD	Rheumatology improved diagnostic confidence and classification.	Confirms the essential role of rheumatology in accurate ILD diagnosis.
Kim et al., 2023 [18]	Prospective single-center cohort (South Korea)	RA patients with and without ILD	Older age, longer RA duration, seropositivity, reduced FVC/DLCO; heterogeneous HRCT patterns.	Supports integrated pulmonary–rheumatologic assessment.
Dubey et al., 2025 [19]	Multicentre patient-experience survey (UK)	RA-ILD patients	Psychological distress, unmet educational needs, inconsistent messaging.	Shows the importance of coordinated communication and education.

## Data Availability

No new data were created or analyzed in this study.
